# Fluorescence-Activated Cell Sorting Analysis of Heterotypic Cell-in-Cell Structures

**DOI:** 10.1038/srep09588

**Published:** 2015-04-27

**Authors:** Meifang He, Hongyan Huang, Manna Wang, Ang Chen, Xiangkai Ning, Kaitao Yu, Qihong Li, Wen Li, Li Ma, Zhaolie Chen, Xiaoning Wang, Qiang Sun

**Affiliations:** 1 Laboratory of General Surgery, The First Affiliated Hospital, Sun Yat-Sen University, 58 Zhongshan er Road, Guangzhou, Guangdong 510080, P. R. China; 2 Laboratory of Cell Engineering, Institute of Biotechnology, 20 Dongda Street, Beijing 100071, P. R. China; 3 The Institute of Life Sciences, the Key Laboratory of Normal Aging & Geriatric, the State Key Laboratory of Kidney, the Chinese PLA General Hospital, Beijing 100853, P. R. China; 4 Department of Oncology, Beijing Shijitan Hospital of Capital Medical University, 10 TIEYI Road, Beijing 100038, P. R. China; 5 Institute of Molecular Immunology, School of Biotechnology, Southern Medical University, Guangzhou 510515, P. R. China; 6 Department of Stomatology, Affiliated Hospital of Academy of Military Medical Science, 8 Dongda Street, Beijing 100071, P. R. China; 7 National Key Laboratory of Medical Immunology and Institute of Immunology, Second Military Medical University, 800 Xiangyin Road, Shanghai 200433, P. R. China

## Abstract

Cell-in-cell structures (CICs), characterized by the presence of one or more viable cells inside another one, were recently found important player in development, immune homeostasis and tumorigenesis etc. Incompatible with ever-increasing interests on this unique phenomenon, reliable methods available for high throughput quantification and systemic investigation are lacking. Here, we report a flow cytometry-based method for rapid analysis and sorting of heterotypic CICs formed between lymphocytes and tumor cells. In this method, cells were labeled with fluorescent dyes for fluorescence-activated cell sorting (FACS) by flow cytometry, conditions for reducing cell doublets were optimized such that high purity (>95%) of CICs could be achieved. By taking advantage of this method, we analyzed CICs formation between different cell pairs, and found that factors from both internalized effector cells and engulfing target cells affect heterotypic CICs formation. Thus, flow cytometry-based FACS analysis would serve as a high throughput method to promote systemic researches on CICs.

For decades, pathologists have seen a type of special structures, characterized by morphologically normal cells enclosed by other cells, in a variety of human tumor samples. Some terms, such as “cell cannibalism” and “cytophagocytosis” and the like, were used to describe these unique structures, which were recently given a unified name “cell-in-cell” structures (CICs)^1^,^2^. Based on the cells involving in structure formation, CICs could roughly be classified into two categories: 1) homotypic CICs, in which structures are formed between cells of same type like epithelial cells; 2) heterotypic CICs, where cells of different types such as epithelia and lymphocytes participate in structure formation^2^.

Researches on CICs became a subject of interests in recent years, largely due to the finding that formation of CICs would lead to the death of majority of the internalized cells^3^,^4^,^5^,^6^,^7^,^8^. Therefore, one process responsible for homotypic CICs formation, entosis, was recommended as a death mechanism by the Nomenclature Committee on Cell Death^9^. Recent progresses have shown that CICs formation played important roles in immune homeostasis^5^ and tumorigenesis^10^,^11^,^12^, and is likely an evolutionarily conserved phenomenon^2^. Mechanistically, CICs formation may reflect the competitive nature of confronted cells^12^, representing a novel mechanism of cell competition^13^.

Boosted interests on CICs call for reliable methods for further investigation. We have previously reported methods for the study of entosis, where homotypic CICs were quantified manually by microscopic observation^14^. While microscopic counting was accepted for quantification of various CICs, homotypic^11^,^15^ or heterotypic^5^,^16^, this method turned out to be relatively subjective and labor-intensive and time-consuming for multiple samples. Flow cytometry provides an ideal technique to quantify cells carrying specified fluorophores in a high throughput manner^17^,^18^,^19^. In light of this, we attempt to develop a flow cytometry-based method for CICs analysis. In this study, we demonstrated that heterotypic CICs, formed between tumor cells and lymphocytes, could be identified and sorted out by fluorescence-activated cell sorting (FACS) method under the condition that cell doublets were minimized before flow cytometry analysis. Furthermore, analysis of CICs formed between different cell pairs indentified an active role of host cells in heterotypic CICs formation, which may revise current view that internalizing cells alone drive CICs formation.

## Methods

### Cell culture and treatment

Cell lines PLC/PRF/5, MCF7, SK-BR-3, RD were purchased from American Type Culture Collection (ATCC, Manassas, VA), and cultured as described^20^. Molt-4, Raji and BxPC3 were kindly gifted by Prof. Ya-jun Guo (The Second Military Medical School, China), and cultured as described^20^. NK92MI cells were gifted from Bin Gao (Institute of microbiology Chinese Academy of Sciences), and were grown in α-Modified Eagle Medium (α-MEM) plus 12.5% fetal bovine serum (FBS) and 12.5% horse serum (Gibco BRL, Carlsbad, CA). Cytokine induced killer (CIK) cells were gifted from Wei-dong Han (Chinese PLA General Hospital), and cultured as described^4^.

### Co-culture experiments

Target tumor cell suspension was stained with 2.5 μM CellTracker Green CMFDA dye (Invitrogen, Carlsbad, CA) for 30 min at 37°C in the absence of serum. Monolayer of the tumor cells were incubated in DMEM with 10% FBS at a density of 3.5 × 10^5^ cells/well in 6 well cell culture cluster (Corning, Union City, CA) for 12 h at 37°C in order to adhere. Immune cells were stained with 10 μl CD45-PE (Beckman Coulter, Brea, CA) for 20 min at room temperature prior to co-incubations with adherent tumor cells by a density of 3.5 × 10^5^ cells/well at indicated time to allow CICs formation. Flow cytometry was gated by unstained/stained effector cells and tumor cells alone, and unstained effector/tumor cells co-cultures as well.

### CICs quantification of Giemsa-stained samples

Unbound cells were removed at the indicated times, adherent cells were washed twice in PBS, and fixed in 4% glutaraldehyde for 20 min at room temperature followed by staining with Giemsa (Jiancheng, Nanjing, China). Heterotypic CICs were quantified by a light microscopy (Olympus Optical Co., Tokyo, Japan). The rate of heterotypic CICs was determined as described^20^ by dividing the number of host cells containing immune cells by total number of tumor cells in a specified field: CICs rate (%) = (number of tumor cells with immune cells/number of total tumor cells counted) × 100. About 800 ~ 1000 tumor cells from random representative fields were counted for each single preparation. Immune cells wrapped at least half-way around by target cells were considered to be internalized. Internalization of multiple immune cells into one tumor cell was counted as one CICs.

### FACS analysis of heterotypic CICs by flow cytometry

Since flow cytometry requires cells of non-adherent, cell suspensions were prepared as followed. After co-cultured experiment, remove the medium, wash cells with Dulbecco's Phosphate Buffered Saline (D-PBS) twice and incubated with 0.25% trypsin/EDTA solution (300 μl/well) at 37°C until cells were dissociated from the bottom of the culture dish. Then added same amount of Hank's Balanced Salt Solution (HBSS) with 20% FBS to quench trypsin. Cells were pelleted at 150 × *g* for 5 min, and re-suspended in 400 μl HBSS with 20% FBS. Then the cell suspensions were filtered through flow injection pipe (BD, Cat: 352035) before acquisition on a flow cytometer. Cell-in-cell structures were counted and enriched on a BD FACScan flow cytometer (FACSAria II) (BD Biosciences, San Jose, CA) equipped with a 15 mW, 488 nm, air-cooled argonion laser and three photomultipliers with band pass filters of 530 nm (FL1), 550 nm (FL2), and 670 nm (FL3). CellTracker Green CMFDA was excited by a blue laser and detected by a 530/30 filter. CD45-PE was excited by a blue laser and detected by a 560/40 filter. FSC and SSC voltages of 121 and 324, respectively, and a threshold of 2,000 on FSC were applied to gate on the tumor cells population. All observations were made in log mode. Data were acquired by using BD CellQuest PRO software (BD Biosciences, San Jose, CA), and analyzed by using FlowJo flow cytometry analysis software (Tree Star, Ashland, OR).

To establish optimal instrument settings to gate and reduce background noise, effector cells and tumor cells alone were used as single negative control, effector and tumor cells co-cultures were used as double negative control, CD45-PE stained effector cells or CellTracker Green CMFDA stained tumor cells were used as single positive control. CICs frequency was calculated by the equation: CICs (%) = (double-positive cells in quadrant Q2/single-positive tumor cells in quadrant Q3) × 100.

### Confocal microscopy

Prepare 300 μl cell suspensions to make cytospin on glass slides in a Cytocentrifuge 7620 (Wescor, Logan, UT) at 400 rpm on high acceleration for 5 min. Cytospins were fixed in 4% glutaraldehyde for 20 min at room temperature. Fixed samples were washed in PBS, applied one drop of antifade reagent with DAPI (Invitrogen, Carlsbad, CA) and mounted on the slides with cover slips followed by sealing with nail oil. Imaging was taken by using an FV1000 laser scanning confocal microscope (Olympus Optical Co., Tokyo, Japan).

### Time-lapse microscopy

Time-lapse microscopy was performed as follows. Tumor cells were grown as monolayer on 30 mm glass bottom dishes to adhere for 4 h prior to adding effector cells. Images were obtained every 2 min for the indicated time courses by FV1000 laser scanning confocal microscope (Olympus) and Olympus Image Browser software (Olympus), with a 40× objective and an environmental chamber to maintain temperature (37°C) and CO_2_ concentration (5%).

### Statistics

All experiments were carried out in triplicate and data are indicated as mean ± SD and more than 400 cells were counted in each experiment. GraphPad Prism (GraphPad Software, La Jolla, CA) was used to plot data generated and carry out statistical analysis. *P* values were calculated by Two-tailed Student's *t*-test, with statistical significance assumed at *P* < 0.05.

## Results

### Two-color assay to analyze heterotypic CICs by FACS

**Figure 1:**
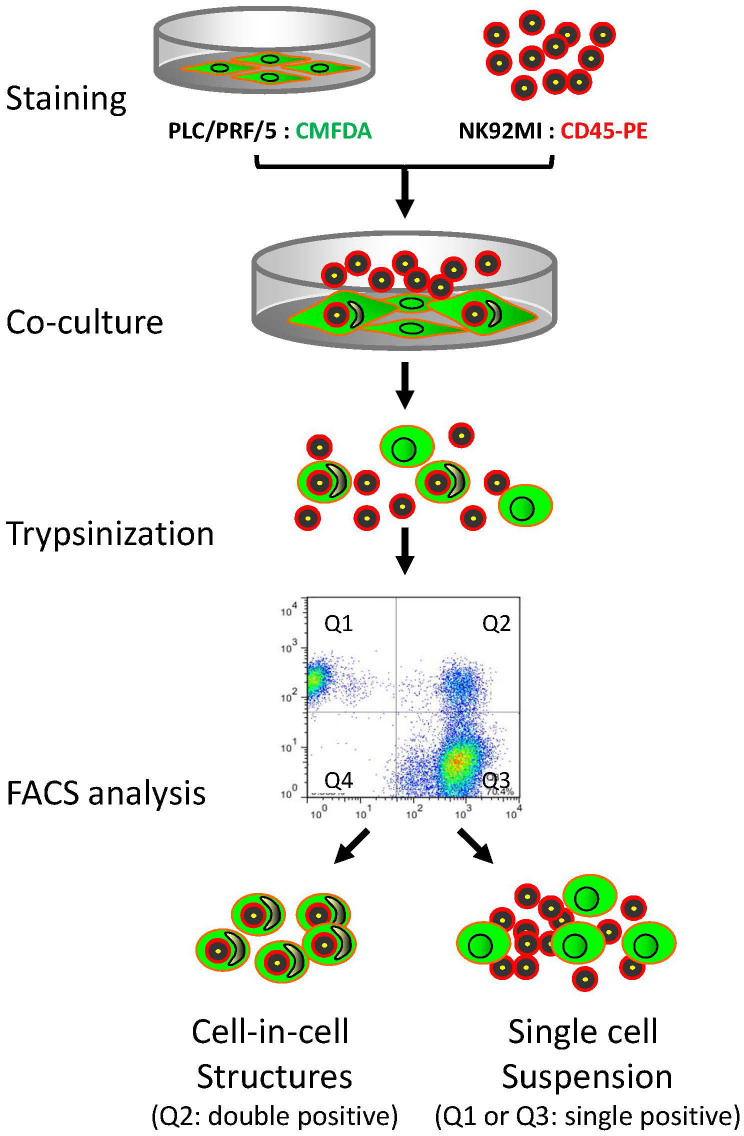
Procedures for FACS analysis of heterotypic CICs. The assay consists of four consecutive steps starting from cell staining, followed by CICs formation and preparing cell suspension, to end up with FACS analysis by flow cytometry. Heterotypic CICs formed between tumor cells and immune cells will be double positive in Q2, while cells in Q1 or Q3 are single positive in red or green, and not involving in CICs formation.

Heterotypic CICs formed between tumor cells and lymphocytes were usually quantified by microscopic counting of Giemsa-stained co-cultures^16^,^20^. Although this method is simple and convenient, the results were sometimes affected by homotypic CICs formed between tumor cells, as it's hard to clearly tell the identity of the internalized cells, especially when they are dying. Moreover, this method was less efficient in analyzing multiple samples and only limited number of cells could be given. One way to solve these problems is to stain the two types of cells in fluorescently different colors and analyze co-cultured cells by flow cytometry. In light of this, we designed a flow cytometry-based method as depicted in Figure 1. Briefly, the lymphocytic effector cells (suspended NK92MI) and epithelial target tumor cells (attached PLC/PRF/5) were stained with CellTracker CMFDA in green and anti-CD45-PE in red respectively before co-culturing for several hours to allow CICs formation. NK92MI cells that don't bind to or penetrate into PLC/PRF/5 cells were washed out prior to preparing cell suspensions for flow cytometry by fully trypsinization. Theoretically, double-positive cells (in Q2) could be read as the heterotypic CICs formed between lymphocytes and tumor cells, while those of single positive are cells not forming CICs. In this way, CICs could be analyzed in a high throughput manner.

### Suppressing cell doublet formation by EDTA, DNase and fetal bovine serum

**Figure 2:**
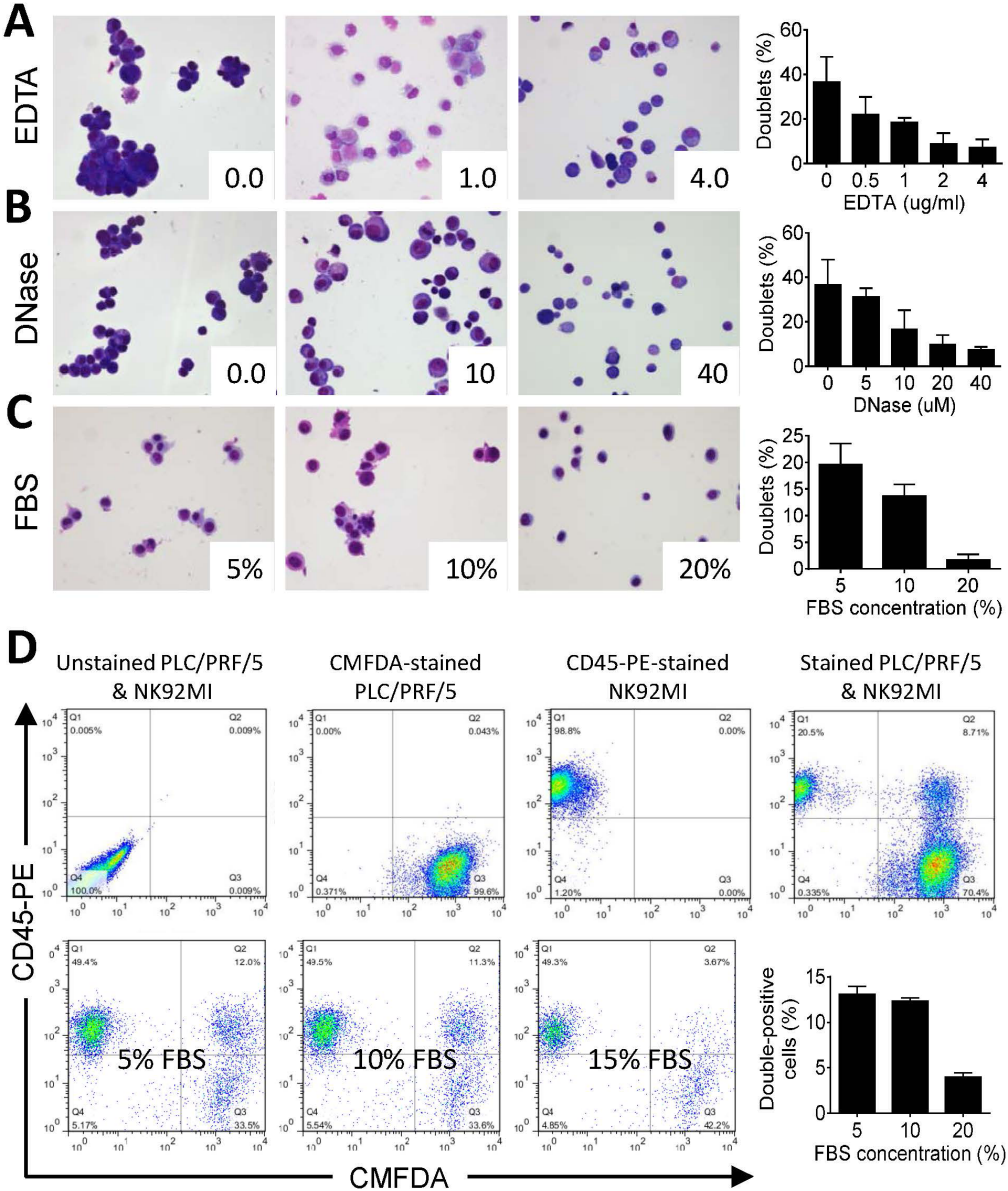
Optimizing factors affecting doublet formation. (A, B, C) Doublet formation in HBSS containing different concentration of EDTA (A), or DNase (B), or FBS (C). Left panels show representative Giemsa images for doublet formation in the presence of different concentration of reagents as indicated. Right panels of column graphs show the quantification of doublet formation. Doublet percentage was calculated as dividing cells in doublets (> = 2 cells) by total cells quantified. More than 10 fields (20× objective lens) were quantified for each experiment. Data are mean ± SD of cells analyzed in triplicate, and are representative of three independent experiments. (D) FACS analysis of double-positive cell population in the presence of different concentration of FBS. Upper panels show gating of controls. Lower left panels show FACS images of double-positive cell population (in quadrant *Q*2) in the presence of FBS (5%, 10% and 20%). Lower right graph shows quantification results of double-positive cell population in the presence of FBS. Data are mean ± SD of four independent experiments.

Our pioneer experiments indicated that flow cytometry couldn't differentiate CICs from double-positive doublets, which sometimes constituted the majority of double-positive population (data not shown). In order to control doublet formation, we optimized the concentration of EDTA and DNase, two factors potentially affecting cell-cell adhension, in HBSS used for cell suspension. As shown in Figure 2A and 2B, although less cell doublet formation was observed in HBSS with higher concentration of EDTA or DNase, considerable amount of cell doublets (about 7% for both) could still form in very high EDTA or DNase condition, which might potentially be toxic to cells and therefore are not ideal for doublet inhibition. Interestingly, we found that doublet formation was efficiently suppressed to quite low level (about 1.5%) when cells were suspended in HBSS with 20% fetal bovine serum (FBS) (Figure 2C), probably due to its ability to block the binding sites for cell-cell adhesion. Accordingly, double-positive cells, including doublets and CICs, decreased drastically from ~12.5% to ~4% with increased FBS concentrations (Figure 2D), indicating the presence of large amounts of cell doublets in double-positive cell population in low-serum medium. A combination of 20% FBS with EDTA and/or DNase didn't give significantly better inhibition of cell doublets (data not shown), therefore, 20% FBS is used for following analysis.

### CICs analysis by FACS correlates well with that by microscopic counting

**Figure 3:**
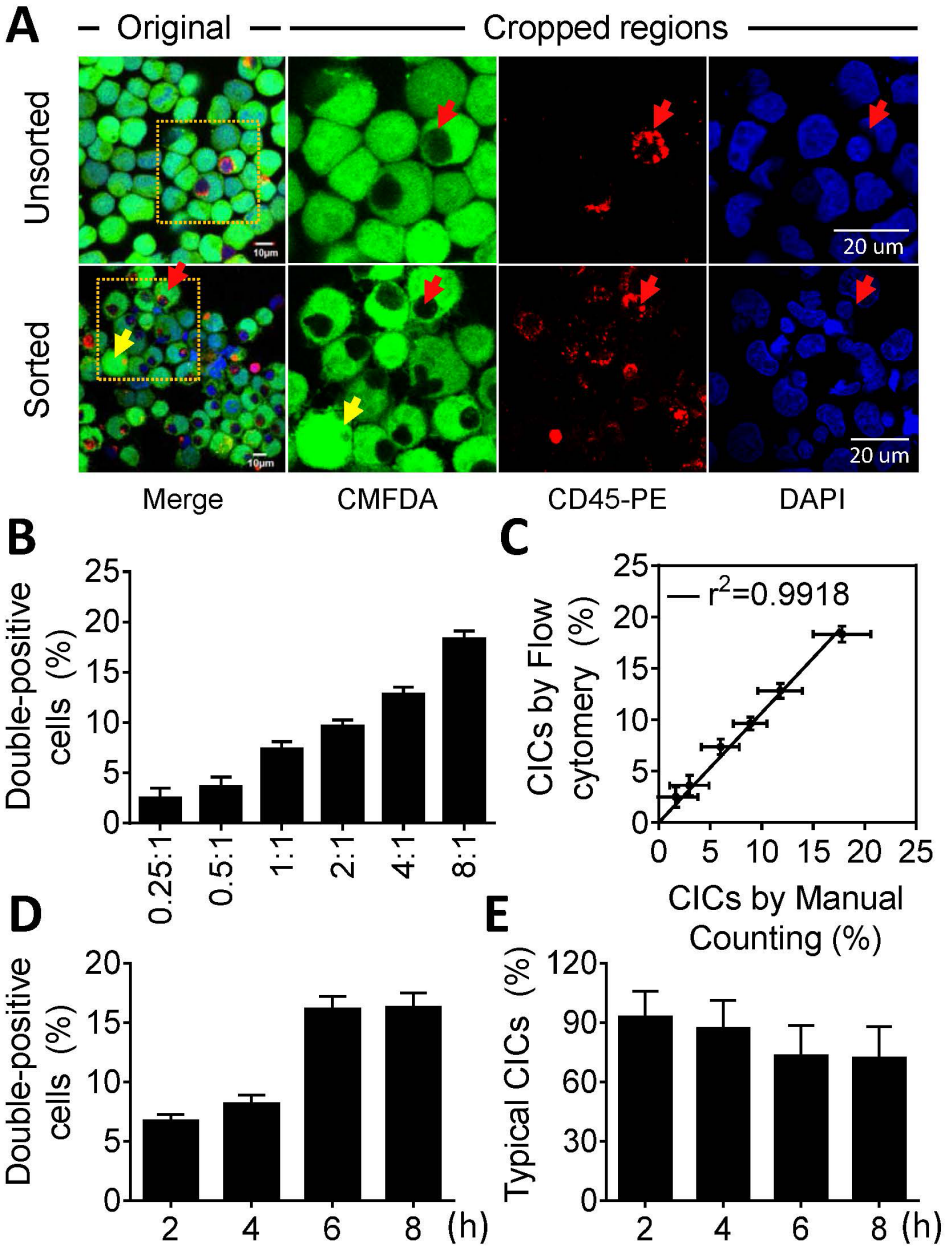
CICs quantified from flow cytometry and manual counting are tightly correlated. (A) Representative confocal images for cytospins of sorted and unsorted CICs. The boxed regions in left images (scale bars: 10 μm) are displayed in three separated channels for PLC/PRF/5 tumor cells (green), NK92MI lymphocyte (red) and nuclei (blue), respectively (scale bars: 20 μm). Red arrows indicate typical CICs; yellow arrows indicate atypical CICs which resulted from death and fragmentation of internalized red cells. (B) Quantification of CICs formed between NK92MI and PLC/PRF/5 of different ratios (NK92MI:PLC/PRF/5) by flow cytometry. Cells were co-cultured for 4 hours. Data are mean ± SD of three independent experiments. (C) Correlation between CICs determined by flow cytometry and by manual microscopic counting. Standard error bars are represented on the horizontal axis for manual counting and vertical axis for flow cytometry counting. Data are mean ± SD of three independent experiments. (D) CICs formed at the various time points as quantified by flow cytometry. Data are mean ± SD of three independent experiments. (E) Percentages of typical CICs in cytospins by flow cytometry enrichment from various time points of co-culture. Data are mean ± SD of CICs analyzed in triplicate, and are representative of three independent experiments.

As described above, high concentration of FBS might be a good candidate to inhibit doublet formation, which will help read out CICs from double-positive cell population. To test this, we performed FACS analysis of cells prepared in 20% FBS. Double-positive cells were sorted out and observed by microscopy. As shown in Figure 3A, CICs were highly enriched after FACS, the typical morphology is that each green cell (CMFDA) is surrounding a black hole filled with red dots (CD45-PE) and blue nucleus (red arrows), an average purity of >85% could be generally achieved with some experiments gave >95% purities (Figure 3E). Moreover, after 4-hours co-culture of different effector:target ratio, the CICs frequencies quantified by FACS (Figure 3B) were tightly correlated (r^2^ = 0.9918) with those from manual microscopic counting (Figure 3C), suggesting CICs constitutes majority part of the double-positive cell population when cells were prepared in 20% FBS HBSS, which is a suitable condition for CICs analysis by flow cytometry.

**Figure 4:**
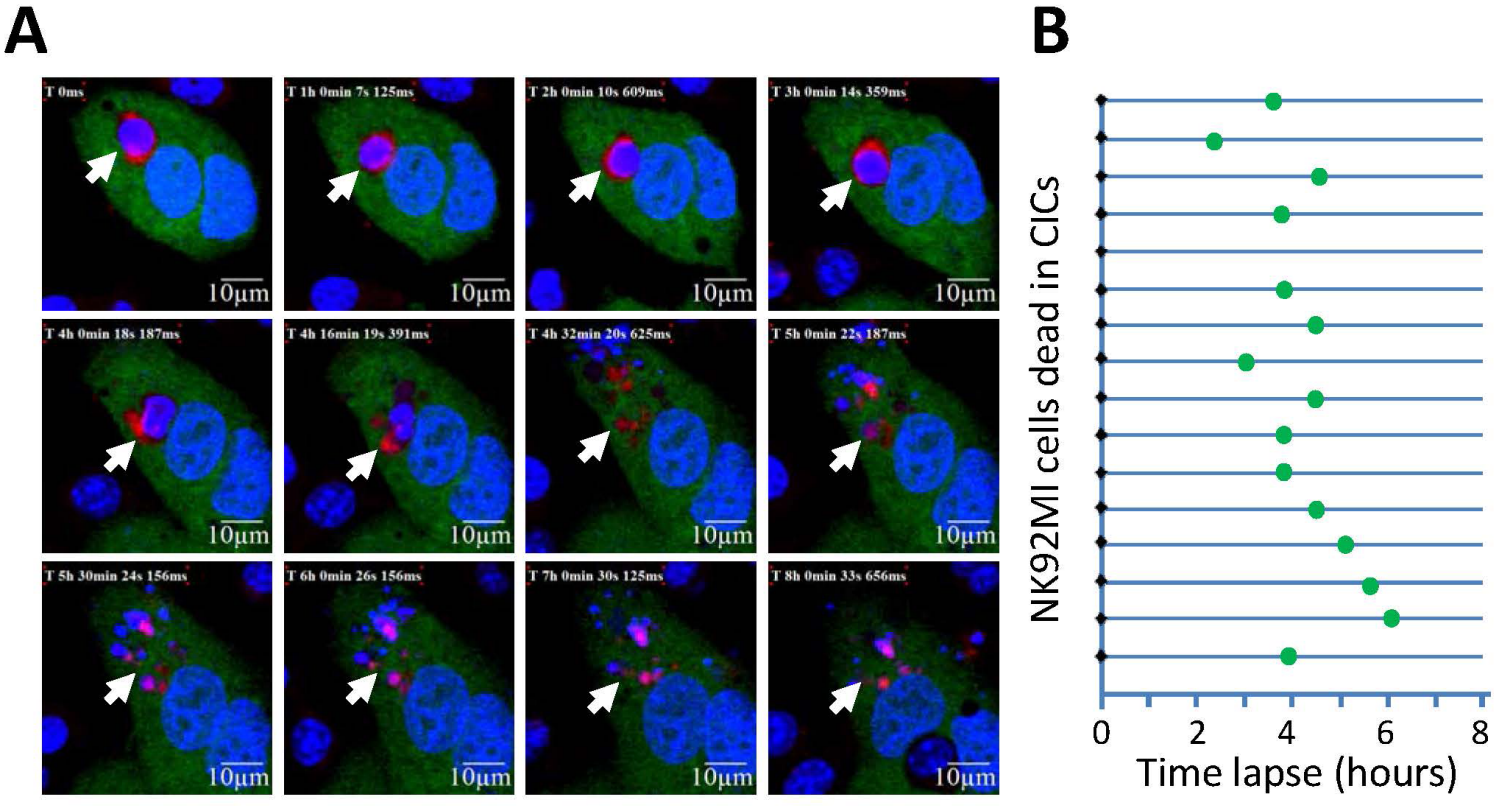
Rapid fragmentation of internalized effector cells. (A) Images captured at different time points show the process of death and fragmentation of NK92MI cells after being internalized. Arrows indicate NK92MI cells or its fragmented corpse. (B) Summary of death events, as indicated by the green dots, of internalized NK92MI cells. Majority of the cell death took place within 6 hours of microscopic time lapse.

Intriguingly, we found that the percentage of CICs with typical morphology among CICs sorted by FACS decreased with longer co-culture (Figure 3E), although the overall frequency increased with time (Figure 3D). We reasoned this for death and fragmentation of internalized effector cells, generating atypical CICs characterized by the presence of pieces of red dots in host target cells (yellow arrows in Figure 3A). In agreement with this, we found, by microscopic time-lapse, that some internalized cells underwent rapid death and fragmentation, morphologically resembling those atypical CICs sorted from flow cytometry, within 6 hours (Figure 4).

### FACS is also applied to analyze other heterotypic CICs

**Figure 5:**
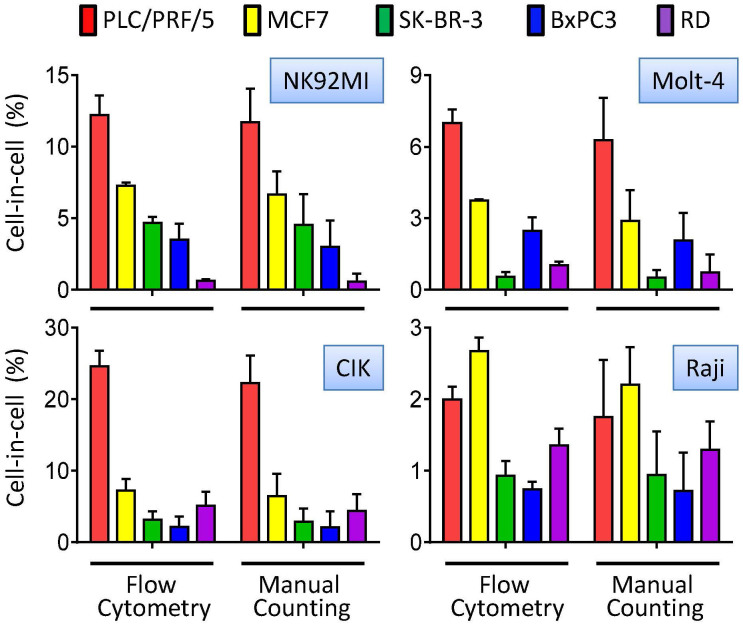
Quantification of CICs formed between different cell pairs. CICs formed between tumor cell lines and immune cells were quantified by flow cytometry and manual microscopic counting, respectively. CellTracker Green CMFDA-stained tumor cells and CD45-PE-stained immune cells were co-cultured for 4 h. Half part of every co-culture was tested by flow cytometry, the other part was quantified by manual microscopic counting. Data are mean ± SD of CICs analyzed in triplicate, and are representative of three independent experiments.

To examine whether the FACS method is also applicable to analyze heterotypic CICs formed between a broad ranges of lymphocytes and tumor cells, we introduce into this assay additional cell lines, including four additional tumor cells (MCF-7, SK-BR-3, BxPC3 and RD) and three more types of immune cells including T cells (Molt-4), B cells (Raji) and cytokine induced killer cells (CIK). Heterotypic CICs formed between them were systemically analyzed by flow cytometry along with manual microscopic counting side by side. In all cases, the CICs quantifications from FACS displayed no significant differences from those by microscopic counting (Figure 5), suggesting FACS by flow cytometry is a reliable method to detect heterotypic CICs formed between lymphocytes and tumor cells. Interestingly, we found that some tumor cells, such as PLC/PRF/5, displayed higher abilities over others to form CICs with immune cells, which suggests that host/tumor cells, in addition to effector/immune cells, also play important role in CICs formation as discussed below.

## Discussion

Although being extensively reported in human tumors for a long time, CICs were short of mechanistic studies until recent years. Several forms of research models had been established, including entosis^3^, emperitosis^4^, heterotypic cell cannibalism (HeCC)^6^, homotypic cell cannibalism (HoCC)^8^, suicidal emperipolesis (SE)^5^ and phagoptosis^21^ and the like. While homotypic CICs formed by mechanisms like entosis and HoCC mainly involve in tumor development and progression^8^,^10^,^11^,^12^, heterotypic CICs formed by emperitosis, HeCC and SE etc. are implicated in broader biological processes, such as development of immune system^22^, immune homeostasis^5^, tumorigenesis^4^,^16^ and neurodegeneration^23^ and the forth. Our previous work showed that heterotypic CICs were actually prevalent in inflammatory tissues^20^. Therefore, it's important for the field to develop an efficient method for accurate and high throughput analysis of heterotypic CICs. In this study, we established a method based on flow cytometry technique for rapid analysis of heterotypic CICs (Figure 1), and conditions for discriminating CICs from cell doublets were optimized to obtain high purity of CICs readout (averagely > 85%) (Figure 2 and 3). In addition, this method was shown to be also useful to analyze CICs of various cell pairs (Figure 5), thus providing a powerful tool for further mechanistic study of heterotypic CICs. It should be noted that traditional microscope-based analysis of CICs could not distinguish partial and complete cell-cell internalizations. As we know, due to cell-cell interaction, many partial cell-cell internalizations do not eventually become real CICs. The method described here utilizes Trypsin to dissociate the cells, under which condition the partial cell-cell internalization will be disrupted. Therefore, this method is helpful to analyzes genuine CICs.

For the assay to be successful, in addition to reducing doublets while maintaining cells viability, particular attention should be paid to the way to label cells fluorescently. The fluorophores must be highly sensitive and not leaky. We use CellTracker Green CMFDA and CD45-PE to stain tumor cells and immune cells, respectively. The advantage of CellTracker Green CMFDA and CD45-PE are their shared stimulation by laser of 488 nm that is more standard across all ranges of instrumentations, including less expensive benchtop models that use solid state lasers. Other dyes such as CellTracker Orange CMTMR and CellTracker Red CMTPX belong to this series of fluorescent probes, but need special excitation wavelength and didn't work well for flow cytometry assay in our hands (data not shown). We choose CD45-PE on the account of its specificity for immune cells and no tumor cell staining. Furthermore, the probe and antibody didn't show detectable influence on cell viability and proliferation which is necessary for further research. Accordingly, the combination of CellTracker Green CMFDA and CD45-PE is recommended for analyzing heterotypic CICs between tumor cells and CD45-positive lymphocytes.

Distinct from phagocytosis of dying cells, CICs formation involves the engulfment of viable cells. Moreover, in most cases, the engulfment was believed to be an active process driven by the internalizing cells rather than the cells being penetrated^3^,^16^. And polarized actomyosin contraction compartmentalized by junctional p190A RhoGAP within internalizing cells, together with epithelial cadherins-mediated adherens junctions, promotes CICs formation in entosis^11^. Nevertheless, the outer host cells were also found playing roles during CICs formation in some cases. For example, metastatic melanoma cells displayed higher ability than primary tumor cells to engulf both dead and live lymphocytes^6^, and Nupr1 down-regulation conferred pancreatic tumor cells ability to engulf their neighbors upon activating TGF-β signaling^8^. In this work, we systemically analyzed the CICs formed between a group of tumor cell-lymphocyte pairs by using the methods described above. Interestingly, we found that lymphocytes differed in their abilities to penetrate different tumor cells to form heterotypic CICs. For example, NK92MI cells could form CICs with PLC/PRF/5 in a frequency of 12% or so in 4 hours, the frequency is less than 1% for RD cells, which is consistent across all the tumor cell-lymphocyte pairs examined (Figure 5). This phenotype could be explained by a role of host target cells (tumor cells) in CICs formation, at least in this work. Taking together our data and those published, we proposed that both internalizing effector cells and host target cells participated in the formation of CICs, which is an integral part of current view that CICs formation is actively driven by the internalizing cells. Future researches on the topic will provide more mechanistic interpretation for this phenotype.
